# Older Adults’ Perceptions About Using Intelligent Toilet Seats Beyond Traditional Care: Web-Based Interview Survey

**DOI:** 10.2196/46430

**Published:** 2023-12-01

**Authors:** Pouyan Esmaeilzadeh

**Affiliations:** 1 Department of Information Systems and Business Analytics College of Business Florida International University Miami, FL United States

**Keywords:** older adults, age tech, intelligent toilet seat, survey interview, qualitative study, mobile phone

## Abstract

**Background:**

In contemporary society, age tech (age technology) represents a significant advancement in health care aimed at enhancing patient engagement, ensuring sustained independence, and promoting quality of life for older people. One innovative form of age tech is the intelligent toilet seat, which is designed to collect, analyze, and provide insights based on toileting logs and excreta data. Understanding how older people perceive and interact with such technology can offer invaluable insights to researchers, technology developers, and vendors.

**Objective:**

This study examined older adults’ perspectives regarding the use of intelligent toilet seats. Through a qualitative methodology, this research aims to unearth the nuances of older people’s opinions, shedding light on their preferences, concerns, and potential barriers to adoption.

**Methods:**

Data were collected using a web-based interview survey distributed on Amazon Mechanical Turk. The analyzed data set comprised 174 US-based individuals aged ≥65 years who voluntarily participated in this study. The qualitative data were carefully analyzed using NVivo (Lumivero) based on detailed content analysis, ensuring that emerging themes were coded and classified based on the conceptual similarities in the respondents’ narratives.

**Results:**

The analysis revealed 5 dominant themes encompassing the opinions of aging adults. The perceived benefits and advantages of using the intelligent toilet seat were grouped into 3 primary themes: health-related benefits including the potential for early disease detection, continuous health monitoring, and seamless connection to health care insights. Technology-related advantages include the noninvasive nature of smart toilet seats and leveraging unique and innovative data collection and analysis technology. Use-related benefits include ease of use, potential for multiple users, and cost reduction owing to the reduced need for frequent clinical visits. Conversely, the concerns and perceived risks were classified into 2 significant themes: psychological concerns, which included concerns about embarrassment and aging-related stereotypes, and the potential emotional impact of constant health monitoring. Technical performance risks include concerns centered on privacy and security, device reliability, data accuracy, potential malfunctions, and the implications of false positives or negatives.

**Conclusions:**

The decision of older adults to incorporate intelligent toilet seats into their daily lives depends on myriad factors. Although the potential health and technological benefits are evident, valid concerns that need to be addressed remain. To foster widespread adoption, it is imperative to enhance the advantages while simultaneously addressing and mitigating the identified risks. This balanced approach will pave the way for a more holistic integration of smart health care devices into the routines of the older population, ensuring that they reap the full benefits of age tech advancements.

## Introduction

### Age Technology

Worldwide demographic trends have witnessed an increase in the proportion of the older adult population. Many older adults have physical conditions or health issues that must be constantly monitored. However, mobility limitations, cognitive impairment, chronic pain, and the costs of being monitored through frequent appointments and admissions may challenge older adults to be engaged in their health care [1]. These issues may negatively affect the functional abilities, physical activities, social activities, and quality of life of older adults. Moreover, aging-related problems may significantly contribute to social isolation and depression [2]. Thus, these physical (eg, declining health and increased vulnerability to diseases), emotional (eg, increased risk of depression and loneliness), cognitive (eg, decline in memory and cognitive abilities), social (eg, reduced social interactions), and economic (eg, difficulty in paying for long-term care) issues may impair independence and the ability to perform daily activities [3].

It is critical to address the combinations of these aging-related issues and find strategies and ways to support older adults as they age to help maintain their independence and overall well-being. Previous studies have highlighted that technology may improve physical, cognitive, emotional, social, and economic issues and help older adults perform core daily activities [4,5]. The development of new technologies and technological devices enables older adults to manage their health conditions effectively, efficiently, and independently. Age technology (age tech) is designed to meet the needs of older adults and those caring for them [6]. Various technologies and devices are devoted to supporting and improving the quality of life for older adults as they age. Age tech can potentially improve the lives of older adults in different ways. First, age tech for physical issues is an assistive technology that helps with mobility and independence [7]. Second, age tech is a health care technology for managing diseases and well-being that provides remote monitoring systems through wearables and devices [8]. Third, age tech aids in daily tasks such as smart home technologies that facilitate activities of daily living using voice commands or mobile devices. Fourth, age tech is used for cognitive functions, such as cognitive aids that help with memory and cognitive abilities, including virtual assistants and reminder apps [9]. Fifth, age tech for emotional and social issues is a communication technology that reduces feelings of loneliness and isolation through videoconferencing and messaging applications [10].

Moreover, studies show that using age tech could potentially help reduce health care costs (to address economic issues) in some ways [11]. For instance, age tech can allow older adults to receive medical care without leaving their homes to visit health care professionals in person or potentially prevent unnecessary hospitalization or readmissions, leading to cost savings. Age tech could potentially lower overall health care costs by improving medication management and helping older adults better manage chronic conditions.

The key areas examined in previous studies on aging technology can be categorized into 6 groups. The first category focuses on demographic trends related to the aging population trends, such as increasing life expectancy and the growing number of older adults worldwide [12]. The second category is related to the impact of technology on aging and how technology can support older people in improving their overall quality of life by facilitating day-to-day activities and ensuring that aging individuals can lead independent and comfortable lives (such as using tools to adjust lighting, room temperature, or lock doors using voice commands) [13]. The third refers to the design and usability of age tech, such as user-centered design, accessibility, ease of use, and usability of technology for older adults [14]. The fourth group considers the impact of age tech on the health and well-being of older people, such as the technology used to manage existing health conditions, prevent potential diseases, or even boost mental health [15]. The fifth category examines the ethics of collecting biological and personal habit data in residences and ethical considerations of using age tech for older adults, such as privacy, security, confidentiality, and potential exploitation [16]. The last emphasizes the future directions of age tech, such as the potential for new personal health devices, artificial intelligence (AI)–powered applications, and emerging technologies to support older adults. This aligns with previous studies highlighting the disruptive types of AI systems being implemented in routine care [17].

### Smart Toilet Seats as a Subset of Age Tech

Using age tech can have several advantages for older adults, such as improved access to care, better health management, increased independence, and connected care [18,19]. However, the older population may not be likely to use age tech because of several potential technological challenges, such as cost, physical challenges, lack of confidence and support, complexity, inadequate infrastructure, and privacy and security concerns [20-22]. A study showed that older people are less willing to modify their housing using technology to meet their needs [23]. As a result, older adults may resist using age tech, preferring traditional methods of receiving health care or communicating with health care givers and professionals [24-26]. Previous studies have examined the adoption of various age tech solutions (eg, personal health devices, wearable technology, telemedicine, health care robots, and social robots) with different functionalities. The adoption of smart technology in the home is on the rise, with internet-connected devices such as smart speakers, appliances, and home automation systems becoming increasingly common [27]. Smart toilets are one area of smart home technology that promises to improve health outcomes. Smart toilets are integrated with sensors and analytical capabilities that can track various health and wellness metrics, from heart rate to weight changes. For aging and disabled populations, smart toilets present an opportunity to monitor health changes unobtrusively and share important data with medical professionals through telehealth platforms.

Smart toilet technology is rapidly gaining popularity, with the global smart toilet market projected to grow significantly in the coming years. The market was valued at US $3.6 billion in 2022 and is expected to reach US $5.5 billion by 2029, witnessing a compound annual growth rate of 6.2% [28]. Another report suggested that the market could reach a value of US $22.2 billion by 2030, with a compound annual growth rate of 15.12% [29]. The increasing demand for advanced bathroom solutions, driven by growing consumer awareness about hygiene and sanitation, is a key factor contributing to this growth [30].

Smart options can reduce strain and effort for caregivers assisting people who require help in using the toilet. With remote operation through a smartphone app, caregivers do not have to be directly present to assist older adults. Beyond basic accessibility features, smart toilets can monitor vital signs, track changes, and share data with medical teams via telehealth platforms. Sensors in smart toilets can measure heart rate and blood pressure when a person sits. Optical sensors can evaluate a person’s vein health to screen for conditions such as congestive heart failure. Smart toilets can also track urine and stool properties such as color, volume, concentration, and frequency to detect possible urinary tract infections, dehydration issues, and gastrointestinal problems. Trends and concerns regarding changes can automatically send alerts to a person’s physician or care team to enable rapid intervention and treatment. Smart toilet seats can detect multiple signs of illness through automated urine and stool analyses. This early detection can help users address health issues before they become more severe, potentially preventing hospitalizations and the spread of infectious diseases among older adult communities [31]. Smart toilet seats have been shown to be beneficial in older adults living in residences. For example, the TrueLoo toilet helped reduce patient falls by 11% in a study involving memory care patients in California [32]. By tracking changes in urine and providing daily wellness reports, smart toilets can aid in the early detection of health issues and provide faster and more efficient care [33].

### Adoption by Older People

The global impact of the COVID-19 pandemic has underscored the critical importance of age tech for the future, especially as it relates to the health care and well-being of the aging population [34]. During the pandemic, many older adults found themselves isolated from their families and usual support systems, leading to an increased reliance on technology. Out of necessity, many older adults adapted to new digital platforms, including telehealth services and communication applications, to stay connected with their health care providers, families, and communities. This unexpected shift brought about a transformation in the way older adults perceive and interact with technology [6]. Their newfound comfort with these platforms may potentially translate into a more receptive attitude toward other innovative solutions and digital services [35]. By drawing parallels with their recent embrace of digital health tools during the pandemic, it becomes evident that there could be growth in acceptance and trust in age tech among the older adult community. This evolving landscape presents a promising opportunity for the wider adoption of smart health technologies tailored to the unique needs of older adults.

The aging population faces unique health challenges and monitoring their well-being is paramount. The human body, through its waste products, provides valuable insights into health and well-being that can be critical for health care. Although this information is rich in diagnostic potential, there has traditionally been a reluctance, especially among older people, to harness these data. This hesitance might stem from cultural taboos, privacy concerns, or simply the lack of user-friendly and noninvasive tools to analyze waste matter [21,22,36]. Understanding this context is imperative for innovative solutions tailored to older adults. Intelligent toilet seats, designed with the consideration of older people in mind, present a groundbreaking approach. By seamlessly integrating technology with daily utility, these devices provide older adults with a nonintrusive method to monitor their health indicators continuously. However, to realize their full potential, it is crucial to address the acceptance of such technology among older adult demographics. It is not merely about proving the technological efficacy of the intelligent toilet seat; it is equally vital to demonstrate its value in a manner that resonates with older people. In a landscape with various health monitoring tools, the justification for an intelligent toilet seat lies in its unique proposition of unobtrusive, continuous health monitoring that respects the comfort and privacy of older people. Addressing this acceptance challenge is vital for the broader adoption and success of such innovations in older adults’ health care.

### Study Objectives

This research focuses on an AI-powered device that has been limitedly studied in the age tech literature. An intelligent toilet seat combines an internet-connected toilet seat and continuous monitoring using AI to generate valuable insights from waste matter discharged from the human body. This advanced technology, mainly used in homes or nursing facilities, seeks to analyze the excreta of individuals to derive valuable health insights from their toileting patterns. We used a qualitative approach in this study as the mentioned age tech is designed based on purposes different from a traditional toilet seat and is still relatively new to the public. Moreover, little is known about older adults’ adoption patterns related to this system in previous studies. Thus, we aimed to gain a deeper understanding of older adults’ perspectives and attitudes regarding a particular age tech (ie, intelligent toilet seats).

The main objectives of this study are as follows:

1. Explore older adults’ perceptions and understanding of intelligent toilet seats and their attitudes toward using them in their daily lives
2. Discover the barriers and challenges older adults may encounter when using intelligent toilet seats
3. Uncover the factors that encourage older adults to adopt intelligent toilet seats
4. Examine the effectiveness of interventions aimed at promoting the adoption of intelligent toilet seats among older adults

This qualitative study could allow researchers to gain a rich and nuanced understanding of the expectations, perceptions, and attitudes regarding intelligent toilet seat adoption among older adults. This information could inform the development of interventions to improve age tech integration into health care services, support user adoption based on the potential benefits and risks of the technology, and identify areas where additional research is needed.

### Literature Review

According to World Health Organization reports, the global population is aging rapidly, with the number of adults aged ≥60 years expected to more than double between 2015 and 2050 [37]. This demographic shift comes with various health challenges, including increased risk of falls, incontinence, and other conditions affecting quality of life and independence. Technology integration into health care, particularly for older people, has been growing. Among these technological innovations, smart toilet seats have gained attention for their potential health monitoring capabilities. Intelligent or smart toilet seats have emerged as an assistive technology that may help address some of these age-related bathroom challenges. This literature review synthesizes the current research on the use and perceptions of intelligent toilet seats among older adults.

The literature review on using smart toilet seats by older adults revealed several key findings. First, the study by Simpson [38] highlighted the use of technologies originally developed for mobile robots, such as smart wheelchairs, to accommodate the population of older people. This suggests that similar technological advancements can be applied to intelligent toilet seats to enhance the experience for aging adults. Pal et al [39] also discussed the negative perception modeling of older adults toward embracing the smart home revolution. This study emphasizes the importance of understanding the attitudes and preferences of older adults when introducing new technologies, including smart toilet seats. Furthermore, using a fuzzy inference system, Kurnianingsih et al [40] proposed a personalized adaptive system for older adult care in smart homes. This research demonstrates the potential for intelligent technologies such as smart toilet seats to be integrated into a comprehensive ecosystem that caters to the specific needs of older adults.

Moreover, Borelli et al [41] present HABITAT, as Internet of Things (IoT) solution for independent older adults. This study highlights the importance of interoperability among different smart devices, which can be applied to designing and implementing smart toilet seats to ensure seamless integration within a larger smart home environment. Finally, Marques et al [42] emphasized the significance of innovative and assistive eHealth technologies for the older adult demographic. This study underscores the potential benefits of incorporating smart toilet seats as part of a broader ecosystem of eHealth solutions to improve the overall well-being of aging adults.

Smart toilet seats are a relatively new technology that integrates sensors, AI, and automation to add advanced features to a standard toilet seat [43]. Key functions of smart toilet seats include the ability to sense the presence of a user, adjust the seat temperature, offer remote control options, analyze toilet use patterns, and, in some cases, monitor basic health data such as heart rate and blood pressure [44]. Along with recent market research reports, major manufacturers such as Toto, Kohler, and Brondell have recently introduced smart toilet models; however, their adoption remains limited [45].

According to Huang et al [46], researchers plan to apply funding to a smart toilet seat model embedded with sensors that can collect vital signs. A recent study explored the development of a smart toilet system for aging people and persons with disabilities [47]. Respondents felt that a smart toilet seat could be beneficial, highlighting its potential for improving comfort, accessibility, and dignity for older adults. Another study concluded that respondents had positive perceptions of smart toilet seats, emphasizing their potential benefits [48]. The literature review in this study also discusses the effects of extended exposure to smart toilet systems. A study presented a smart AI-based toilet concept that uses 3D depth data to automatically preconfigure the height and tilt of a motorized toilet seat [49]. Although not specifically addressing the needs of older adults, such features can enhance accessibility and autonomy for users with mobility issues. An article discussed a disease-detecting “precision health” toilet that can sense multiple signs of illness through automated urine and stool analysis [31]. Although not focused on older adults, this technology has the potential to benefit users of all ages by providing early detection of health issues. Some smart toilet seat models also analyze urine and stool to screen for possible health issues, such as urinary tract infections, kidney disease, and colorectal cancer [50]. Although still in the early stages, such capabilities could provide convenience and peace of mind for older users and caregivers.

Although intelligent toilet seats show promise for older users, research points to several limitations and concerns. Privacy and hacking risks are frequently raised as smart seats collect personal use data [33]. The seats’ high costs, complex interfaces, and need for regular cleaning may also deter adoption, especially among older people without caregiver support. Studies further indicate reluctance among older adults to use new bathroom technologies. Yeo et al [51] conducted focus groups with adults aged >65 years and found broad skepticism; respondents felt that smart toilets were unnecessary, embarrassing, and too complicated to use. This highlights the need for better education and training regarding the benefits of smart seats.

## Methods

### Research Context

This study focuses on a particular age tech—intelligent toilet seats. This age tech combines an internet-connected toilet seat and a continuous monitoring system to generate important insights from excrement and urine. The in-toilet technology measures various parameters of excreta, such as volume, consistency, frequency, color, and chemical composition. The data collected from these toilet seats can be used for various purposes, such as tracking bowel movements for medical diagnosis, monitoring hydration levels, and detecting changes in health status. The key idea is to monitor the early signs and signals of chronic issues that are very common in the older population (or even younger people) and be able to prompt corrective actions before they become more problematic. It is also equipped with sensor technology to determine who the user is and provide results for each user. Scanning the toilet bowl to determine the excreta size and shape, the system can indicate potential health issues such as dehydration, dietary imbalances, gastrointestinal diseases, infections, or even early signs of colorectal cancer. Regular monitoring and analysis of stool and urine can provide insights into an individual’s microbiome health, hydration levels, and kidney function. In addition, any sudden or drastic change in excretion patterns can serve as an alert for the need for medical consultation or intervention.

The smart toilet can report the collected data from toileting logs and send data or analysis of wellness parameters to the designated care team’s dashboard. The data are analyzed using AI, and if abnormalities are detected, the system will send an alert to health care providers’ systems. Smart toilets generally connect with the broader network of interconnected devices, known as IoT, allowing access to the gathered data and additional functionalities. By checking health updates from the system, health care teams can directly inform the user, family, or caregivers of any anomalies or deviations detected in the health data collected by the smart toilet seats. On the basis of the results, health care professionals can provide real-time feedback and suggestions to improve personal health and wellness. The smart toilet can also directly send health data or analysis to users, enabling older people to continuously monitor wellness parameters and health conditions and track trends and insights.

### Type of Smart Toilet Seats

In the rapidly evolving field of smart health monitoring, myriad brands and types of intelligent toilet seats have emerged, each with distinct capabilities and features tailored to specific health requirements. Several basic seats provide comfort, such as warm seats and cleaning functions. Some seats might focus on advanced sensors to detect hydration levels, whereas others can prioritize temperature monitoring or even microbiome analysis. However, our study, in particular, focuses on a specific category of smart toilet seats that primarily analyze excreta, both stools and urine. The rationale behind this focus is the rich array of health insights that can be gained from such waste matter. For instance, variations in stool consistency can indicate digestive health, potential infections, or even chronic conditions such as irritable bowel syndrome. Similarly, urine can offer clues regarding hydration, kidney function, and the presence of specific compounds or infections.

To illustrate, brand A’s intelligent toilet might emphasize pH monitoring in urine to identify potential urinary tract infections, whereas brand B’s product could prioritize stool consistency analysis to detect digestive anomalies. In contrast, the type of intelligent toilet seat we focus on combines both these functionalities, offering a holistic overview of an individual’s health through dual analysis. Although it would be beneficial to provide a comprehensive comparison of all available smart toilet seats in the market, such a broad scope could dilute the primary objective of our study. Our focus remains on understanding older adults’ perception and acceptance of technology that analyzes both stool and urine to detect potential health issues and monitor critical health parameters.

### Study Design

This study used a qualitative research approach via a survey interview. Our study aimed to explore and understand older people’s views about intelligent toilet seats and the factors influencing their feelings, perceptions, and attitudes. As the use of this technology is increasing, there is a need for a solid adoption pattern. Our research’s present state is exploratory and discovery to uncover factors affecting older people adopting smart toilet seats. Thus, leveraging a qualitative approach is appropriate to effectively capture the influential factors that may facilitate or deter adoption in the future [52]. Survey interviews were used as the data collection method in this study. This approach allows researchers to ask a set of standardized questions to a sample of respondents to gather information and collect insights into their opinions, attitudes, behaviors, or experiences [53]. We administered a web-based survey questionnaire to the respondents to obtain more information from the participants by capturing their words, ideas, and expressions. The reason for choosing web-based versus in-person data collection was 2-fold: (1) having in-person interviews with older people could be difficult and inconvenient for them (eg, owing to health conditions) and might interrupt data-collection flows; (2) the web-based survey interview is helpful for conducting social science studies to gather data from larger samples [54].

This research methodology was designed based on content analysis and thematic analysis. In the context of our study on older adults’ perceptions of intelligent toilet seats, both content and thematic analyses offer valuable frameworks. Although content analysis helps structurally categorize and quantify responses, thematic analysis delves deeper into the nuances and underlying patterns within those responses. Both methods, when combined, provide a comprehensive understanding of older adults’ attitudes, beliefs, and potential barriers or facilitators to adopting such technology. This process involves collecting data, coding the data to detect themes and patterns, and using the coded data to develop a theory that explains the relationships between concepts. The baseline questions used in this study focused on 5 categories. The first area was general familiarity with AI and awareness of smart devices. The remaining 4 questions were mainly asked to discover the respondents’ perceptions of the specific age tech being studied: perceived benefits and advantages, perceived concerns and risks, overall opinions, and willingness to use. Multimedia Appendix 1 presents the survey questions used in this study.

At the beginning of data collection, the system was clearly defined, and the features were explained using words and terms that someone not specialized in this field or unfamiliar with this technology could easily comprehend. We also consulted 2 well-published scholars in the field of AI in health care to ensure that the description of the technology being studied was readable and understandable. We made minor changes to the definition and feature description according to the feedback received. We refrained from using phrases or expressions that may convey positive or negative connotations to minimize possible leading effects on the answers. Finally, we conducted a pilot test involving 25 randomly selected older adults to verify the clarity of the survey language. The feedback from these respondents confirmed that they comprehended the study’s aims and the definitions and questions presented to them.

### Ethical Considerations

Beyond obtaining ethical clearance from the institutional review board of Florida International University (Reference #112755), several steps were ensured to maintain the research’s integrity, confidentiality, and ethical soundness. First, participants were provided with a clear and detailed informed consent form. Second, participation was voluntary, and respondents could opt out at any stage without consequences. Third, data anonymity and confidentiality were upheld as no personal identifiers were collected, and data were stored securely with access restricted to research team members. Fourth, the potential risks and benefits of participation were clearly outlined in the consent form.

### Respondents, Data Collection, and Sample Representativeness

Individuals (as potential users of intelligent toilet seats) are the unit of analysis in this study. However, this study focused on older adults who may need to monitor their health and wellness parameters more frequently than other age groups. Older adults may experience various changes that increase the risk of health problems such as diabetes and chronic kidney disease. Monitoring health parameters (such as infection and kidney function) can help detect these issues early and prevent complications. Early detection and treatment can improve health outcomes and quality of life in older people and help physicians identify any changes in health status and adjust treatment plans accordingly. Thus, older people can be the best target market for this age tech.

Two inclusion criteria were set that were consistent with the study objective. The first was age, including older adults aged ≥65 years, and the second was the location, limiting respondents to individuals in the United States. Data collection was performed in January 2023 through Amazon’s Mechanical Turk (MTurk). Previous studies have proven that MTurk is a suitable survey tool for collecting individual-level data [55,56]. Researchers, as requesters, can use this crowdsourcing website to reach out to potential participants (ie, MTurk workers) in numerous countries to conduct a survey. MTurk workers, often referred to as Turkers, are a diverse group of people from across the globe who engage in the tasks known as human intelligence tasks. Several studies compare MTurk to conventional data collection methods in health and medical literature and support using this platform for various academic purposes (eg, in health care research) [57]. Existing literature in clinical research highlights that because of a larger network of people, the MTurk population is more representative of the US population than other web-based surveys [58]. Moreover, some studies validated crowdsourcing platforms (such as MTurk) for collecting data from older adult respondents [59,60].

Although MTurk provides a diverse pool of respondents, it is essential to acknowledge the potential limitations concerning its representativeness. Our sample from MTurk comprises a subset of the older adult population, particularly those familiar with and who have access to technology. This may mean that they are more technology-savvy and possibly more open to adopting new technologies than the general older adult population. Moreover, MTurk workers might have a different socioeconomic status [61]. Participation in MTurk often serves as a supplementary income source, which might indicate a different economic stratum compared with other older adults. Finally, although our study was limited to respondents in the United States, MTurk workers can come from diverse geographical and urban or rural backgrounds. Their distribution might not align perfectly with the broader distribution of older adults in the United States. Thus, in our analysis, we were cautious about overgeneralizing our findings, emphasizing that they primarily apply to older adults familiar with web-based platforms.

All questions were structured and open ended, allowing the respondents to express their in-depth opinions and ideas to generate a more profound understanding of the phenomenon. It should be highlighted that the survey was anonymous, and no personally identifiable information was collected from respondents. We also did not define character limits for answers to avoid fake or false responses owing to the imposed pressure of reaching a data-entry limit. However, we discarded records with no or irrelevant responses. A total of 202 individuals who met the inclusion criteria attempted the survey interview. We found that the responses of 28 individuals were not satisfactory (either no or unrelated responses were provided). Therefore, the final data set included responses from 174 older adults living in the United States. All the respondents received US $5 as an incentive for completing the survey.

### Data Analysis

Following our qualitative method, we used content analysis to interpret and analyze the response transcripts to identify and induce patterns, themes, meaningful structures, and trends, which can help researchers make inferences about the studied population. Content analysis is mainly used to draw inferences about the attitudes, beliefs, or behaviors of the people who produced the content [62]. Two researchers with experience in age tech and older adults’ adoption of personal health devices coded the responses to perform explorative content analysis. The coding procedures used in our qualitative content analysis were open, axial, and selective coding [63]. In the initial coding stage (ie, open coding), researchers independently examined the answers and generated a list of codes or labels that described the content based on their interpretation of the data. The relationships and connections between the previously generated codes were explored using axial coding. At this stage, the codes were grouped into categories and subcategories to explain the relationships between the codes. In selective coding, researchers focused on the most important or relevant categories or conceptual themes that had emerged from the open and axial coding stages.

The 2 coders independently coded line-by-line answers from 174 surveys using the NVivo software tool. With NVivo, researchers imported the data, applied codes, and categorized data into themes. As 2 coders coded the same data, we used interrater reliability (IRR) to measure the degree to which different coders agreed on data coding [64]. We used 3 measures to calculate IRR: percentage agreement, κ coefficient, and intraclass correlation coefficient (ICC) [65]. As discrepancies in coding interpretations arose, it was crucial to ensure that any biases or misunderstandings were addressed. For conflicts in coding, the 2 coders initiated in-depth discussions to explore the roots of their discrepancies, share insights from their individual interpretations, and converge on a shared understanding. This iterative process ensured that both coders fully understood each other’s perspectives and was essential for refining and solidifying the coding framework. Although incorporating a third reviewer might have added another layer of arbitration, we found that our systematic discussions and the use of IRR measures, especially after repeated coding, provided robust validity. The coders’ consensus meetings were comprehensive, focusing on understanding the essence of each response and reconciling the differences. We finalized our coding after reaching a mutual agreement and achieving significantly improved IRR metrics. It is worth noting that the achieved IRR values, after discussion, were well within the acceptable range, demonstrating the reliability of our dual-coder system. We obtained a percentage agreement of 72%, a κ coefficient of 0.69, and an ICC of 0.67. To attain a higher threshold and ensure the validity of the results, we repeated the coding process until the 3 metrics fell within the acceptable range. To achieve better consistency and agreement between ≥2 raters, the coders met to resolve coding disagreements and reach a coding consensus. Then, the coders achieved a percentage agreement of 91%, a κ score of 0.80, and an ICC score of 0.83, representing the validity of the coding process and the results.

## Results

### Demographic Characteristics

The web-based survey was completed by 174 respondents aged ≥65 years. Of the respondents, 73 (41.9%) were aged between 65 and 70 years, 59 (33.9%) were aged between 70 and 75 years, 30 (17.2%) were aged between 75 and 80 years, and 12 (6.9%) were aged between 80 and 85 years. A small majority of the respondents were male, with 92 (52.9%) male and 82 (47.1%) female respondents. A significant proportion of the respondents had undergraduate or graduate education (120/174, 68.9%), indicating that the sample is relatively educated compared with the general population. This is consistent with previous research indicating that individuals with higher education levels are more likely to seek web-based health information and are more aware of technological changes [66,67]. Chronic health conditions were reported by 37.9% (66/174) of the respondents, with diabetes, arthritis, and chronic kidney disease being the most common issues cited. Most of the respondents (127/174, 73%) lived with family or others, whereas the remaining (47/174, 27%) lived alone.

At the beginning of the survey, the respondents were asked about their overall familiarity with AI-powered and smart devices. Overall, 13.8% (24/174) of the respondents indicated that they were not familiar with AI and smart devices, 37.2% (64/174) reported being aware of AI-based devices, though they had never personally used them, and 23% (40/174) were familiar with and had used some general AI-based devices (such as smart thermostats, smart cameras, and robot vacuums). Finally, 46% (80/174) of the respondents indicated familiarity with AI-powered health care tools or devices (such as AI for cancer diagnosis, tumor detection, or managing diabetes). Moreover, 25.3% (44/174) of the respondents reported using AI-enhanced applications to manage chronic health issues and dietary preferences or to receive medication reminders.

Moreover, before sharing the description of smart toilet seats, we asked the respondents whether they knew anything about this technology. Overall, 60.1% (106/174) of the respondents said they had heard about it or read (at least) an article about this topic on social media, newspapers, or websites. After showing the description, all respondents reported that they reasonably understood what a smart toilet seat looked like and how it worked. It is worth noting that none of the respondents directly had used an intelligent toilet seat. This evidence is expected because understudied technology is still relatively new to the older population.

When asked for their overall opinion on smart toilet seats, a large majority, 83.9% (146/174), exhibited positive attitudes, whereas 16.1% (28/174) held negative views toward this age tech innovation. As for their willingness to use this technology, 64.9% (113/174) of the respondents stated that they were willing to use it, 28.2% (49/174) of the respondents considered future use, and 6.9% (12/174) of the respondents were not inclined to use it. Regarding the best use case for adopting this technology, 63.8% (111/174) of the respondents suggested that nursing homes were the most appropriate setting, followed by rehabilitation centers (44/174, 25.3%) and personal use in homes (19/174, 10.9%).

### Positive Opinions (Benefits and Advantages)

Respondents were asked to describe their opinions on the benefits and advantages of using intelligent toilet seats. Textbox 1 shows open codes and common concepts related to the use of age tech. Open codes were derived directly from the raw data, capturing specific details and nuances from respondents’ responses. During open coding, researchers label and categorize phenomena found in the text based on their properties and dimensions. These codes were then grouped by researchers based on shared themes or ideas, capturing the overarching essence or underlying patterns of the data presented. Through this process, the intricate details of individual responses are distilled into broader concepts that capture the collective sentiment and insights of the respondents.

**Open codes for perceived benefits and advantages related to intelligent toilet seats.**

**Early detection of diseases**

- Detection of infectious diseases, anomaly detection, preventive care, timely discovery of serious issues, identification of diseases, preventing serious illness

**Multiple users**

- Many users, usable to several users, supporting multiple users, accessible to different users, multiple individuals

**Easy to use**

- User-friendliness, easy-to-use technology, simple, intuitive controls, no complicated steps, no learning required, no instructions

**Health care cost reduction**

- Lowering the overall cost of health care, reducing costs of travel, lowering the number of appointments, minimizing unnecessary visits, cost of extra treatments

**Seamless connection to clinical reports**

- Interactive communication, real-time data sharing, better connection, sharable reports, better collaboration with caregivers

**Constant monitoring**

- Regular check-ups, ongoing screening, continuing surveillance of health status, monitoring symptoms, constant observation of signs, being controlled

**Safe technology**

- No harm, not dangerous to individuals, safe function, reliable technology, no injuries, no side effects

**Unique function**

- Innovative technology, new functionality, unique attributes, competitive advantage

Table 1 shows 8 categories of constructs. Table 1 also exhibits example quotes selected from the respondents’ answers to the survey questions. These quotes can support the constructs (related to perceived benefits and advantages) extracted from the answers. The 8 constructs are as follows:

1. *Early detection of diseases*: Respondents expressed the view that smart toilet seat technology might offer potential benefits in identifying health issues such as urinary tract infections. They believed that the early identification of these problems would enable users to address them promptly. Some respondents felt that this could prevent more severe health complications such as the spread of infectious diseases within older adult communities. Several respondents highlighted the potential of such technology in facilitating timely and preventive care, thereby possibly enhancing the quality of health care they receive.
2. *Multiple users*: Respondents mentioned an appreciation for the device’s sensor technology, which they understood could identify individual users. They highlighted that, according to their understanding, the system could analyze excreta by assessing attributes such as size, color, consistency, frequency, and shape, allowing for individualized results. This feature, they felt, ensured that multiple residents could use the seat without data overlap. In addition, some respondents noted the potential convenience of suspending data collection, especially during visits from guests, by simply pressing a button on the seat.
3. *Easy to use*: Respondents perceived that the smart toilet could be user-friendly. They believed that it required minimal setup, had an uncomplicated design, and would not require intricate instruction manuals or guidelines. Some respondents understood that the toilet used computer vision technology to gather health data autonomously and to forward it for assessment. As such, many felt relieved that they would not have to engage in maintenance, manually transmit data, or decipher analytical reports. The overarching sentiment was that adopting this intelligent device would not necessitate intricate technological training or education.
4. *Health care cost reduction*: Respondents expressed the belief that maintaining an automated electronic record of bowel movements and urination could potentially lead to financial savings. They felt that such records could minimize the need for regular physician visits, associated transportation costs, and subsequent treatments. Many respondents perceived that smart toilet seats might diminish the expenses tied to health monitoring by curtailing the frequency of physician visits and unwarranted medical examinations. There was a shared sentiment that this technology could be cost-effective in the long term, particularly for older adults grappling with chronic health issues.
5. *Seamless connection to clinical reports*: Respondents noted the potential of the smart toilet to relay data from toileting logs directly to the dashboards of designated care teams. They mentioned that in the event of detected anomalies, direct communication could be initiated with the patient, their family, or even their physician. Furthermore, the option to provide printouts of the data to both the patient’s family and medical professionals was considered valuable. Respondents felt that such features could foster improved collaboration and interaction between caregivers, health care providers, and family members.
6. *Constant monitoring*: Respondents believed that smart toilet seats might offer them a means to consistently oversee wellness indicators and health states, allowing for the identification of trends and insights. They noted the potential for the technology, in collaboration with health care teams, to consistently monitor for any significant health deviations or irregularities without requiring their active participation. Many respondents shared a sentiment of reassurance, feeling that their clinical data would be regularly assessed and any chronic health concerns would remain under the watchful eye of their health care professionals.
7. *Safe technology design*: Respondents highlighted the appeal of smart toilet seats owing to their lack of need for battery charging or wearing additional devices such as pendants. They also emphasized the absence of harmful radiation or potential safety hazards associated with their use. The simplicity of the device, with fewer components that might malfunction, was observed to reduce apprehensions about possible user errors. Many respondents believed that smart toilet seats presented a low-risk tool for older adults in terms of physical safety and potential software issues, which they perceived would not compromise their health.
8. *Unique function*: Respondents were intrigued by the distinctive functionality of the technology, which integrates an internet-enabled toilet seat with continuous monitoring capabilities to derive insights from fecal and urinary outputs. They noted that age tech taps into an emerging scientific domain that blends optics, AI, sensors, IoT, and cloud computing to craft a personalized health portrait based on toileting data. Many respondents felt that such technology shifts the health care paradigm, encouraging a more proactive stance than a reactive one. The bespoke features of this age tech seemed particularly suited to the needs of older adults, especially those managing chronic health conditions.

It should be noted that although both early detection of diseases and constant monitoring aim to optimize health management, they operate on distinct principles. Early detection focuses on the proactive identification of specific health conditions, often before symptoms manifest, aiming for timely intervention when diseases are most treatable. It is rooted in intentionality, targeting diseases known to benefit from early intervention through periodic screenings. In contrast, constant monitoring is an ongoing surveillance of an individual’s overall health status. It encompasses a broader spectrum of health metrics, capturing data continuously, often through technological aids. Its primary purpose is to track and respond to any health changes, whether indicative of a disease or a temporary fluctuation. Although both are crucial, they offer unique approaches and benefits in health care.

**Table 1:** List of constructs, definitions, anecdotal evidence, and count for perceived benefits and advantages related to intelligent toilet seats.

Axial codes (constructs)	Definition	Sample quotes	Respondents, n
Early detection of diseases	The extent to which intelligent toilet seats can improve the chance of early detection of serious health conditions and diseases	“This technology is good for general health checking and early disease detection.” “At my age, any head start on detecting a health problem can make all the difference. If this seat can give me a heads-up about potential issues, then I’m all for it.”	102
Multiple users	The extent to which different individuals can use intelligent toilet seats without possible disruption or confusion	“Me, my wife, or even our kids can use the same smart seat without worrying about result confusion.” “Our household has six people; it’d be great if the intelligent toilet could differentiate between all of us and still function effectively.”	63
Easy to use	The extent to which using intelligent toilet seats is free of effort and does not need training or complicated instructions	“It seems very easy to use this seat because there is no training or complicated instruction on how to use it.” “If it’s called ‘intelligent,’ I assume it’ll be smart enough to make things easy for me.”	81
Health care cost reduction	The extent to which using intelligent toilet seats can reduce overall health care costs.	“I am fed up with many appointments that I need to have to just check my urination and digestion issues. They are unnecessary money going out of my pocket. This seat can solve cost-related issues.” “Preventive care is always cheaper than treating advanced conditions. I see the potential in these toilets to keep me ahead of any health troubles.”	46
Seamless connection to clinical reports	The extent to which using intelligent toilet seats can help users access their clinical data and improve communication and collaboration with caregivers	“I can see my health conditions and clinical reports. Also, my doctors can check out the results promptly. So we can have a better dialogue about my conditions.” “The ability to link my daily health metrics from the toilet to my medical records? Now, that’s smart technology.”	78
Constant monitoring	The extent to which using intelligent toilet seats can enhance the monitoring and screening of wellness parameters	“I believe continuous monitoring of toileting will be useful in the sense that I am sure my wellness is monitored and in case of immediate treatment, I am in good hands. “I think the beauty of this technology is its consistency. The constant monitoring means no anomalies go unnoticed.”	83
Safe technology design	The extent to which intelligent toilet seats have safe design and harmless functionality, and using intelligent toilet seats will not impose physical harm on individuals	“No parts or functionality of these seats can harm my health.” “It appears that the design is foolproof and there’s no risk of any harm, then why not? I’m on board.”	56
Unique function	The extent to which intelligent toilet seats leverage unique and innovative technology for data collection and data analysis	“This technology is very new. Using computer algorithms and AI to detect diseases from bowel movement is very innovative.” “It’s not just about collecting data but how it’s analyzed. The technology in this toilet seems to think out of the box.”	39

Table 2 presents the key themes representing the main perceived benefits and advantages of using intelligent toilet seats. The core themes are health care-related benefits, technology-related advantages, and use-related benefits. The theme of health care-related benefits refers to the contributions of intelligent toilet seats to the early detection of diseases, interactive communication with caregivers, and monitoring of wellness parameters. Technology-related advantages reflect the safe functionality and innovative features of this age tech. The use-related benefits theme describes how smart toilet seats are easy to use, straightforward, efficient, and usable by multiple individuals.

**Table 2:** Selective codes representing perceived benefits and advantages related to intelligent toilet seats.

Selective codes (themes)	Constructs involved	Definition
Health care-related benefits	Early detection of diseases plus seamless connection to clinical reports plus constant monitoring	The extent to which older people may believe that intelligent toilet seats can help detect diseases in their early stages, strengthen interactive connections to clinical data, and maintain constant monitoring of health parameters
Technology-related advantages	Safe technology design plus unique function	The extent to which older people may believe that intelligent toilet seats are safe technology with innovative features
Utilization-related benefits	Easy to use plus multiple users plus health care cost reduction	The extent to which older people may believe that intelligent toilet seats are easy to use, usable by several users, and efficient

### Negative Opinions (Concerns and Risks)

Respondents were asked to describe their perceptions and opinions regarding the concerns and risks of using intelligent toilet seats. Textbox 2 depicts open codes and common concepts related to using this technology.

**Open codes for perceived concerns and risks related to intelligent toilet seats.**

**Embarrassment**

- Feeling embarrassed, uncomfortable, ashamed, unpleasant, discomfort

**Unnecessary concerns**

- Consistent stress, overthinking, obsession, feeling phantom alerts, persistent notifications

**Aging-related stereotypes**

- Negative images, untrue ideas, stereotypes, beliefs not based in fact, negative attitudes

**Privacy and security risks**

- Collection practices, security measures, sharing mechanisms, leaked data, data breaches, hackers, vulnerable systems, data protection

**Data quality risks**

- Invalid and unreliable wellness parameters, malfunction, false negative or false positive, biased results, data accuracy

**Internet outage risks**

- Down network, internet connection problems, Wi-Fi issues, disrupted communication, connectivity issues, slow network, equipment failure, outage

Table 3 demonstrates 6 categories of the constructs. Some sample narrations chosen from the respondents’ answers are presented in Table 3. These quotes can support the constructs (related to perceived concerns and risks) taken from the answers. The 6 constructs are as follows:

1. *Embarrassment*: Respondents expressed potential feelings of discomfort associated with the intimate nature of data collection using the intelligent toilet seat. The primary function of the tool is to analyze fecal and urinary outputs to provide health insights, and this direct focus on personal waste might evoke feelings of embarrassment among older adults. Although the technology operates discreetly, several respondents indicated a preference for a device that monitors wellness parameters in a less intrusive manner.
2. *Unnecessary concerns*: Respondents voiced concerns about the potential for heightened anxiety stemming from continuous monitoring by intelligent toilet seats. Although the technology aims to provide proactive health insights, some older adults felt that being constantly monitored might lead them to become overly preoccupied with minor health anomalies that might not necessarily require medical intervention. The constant anticipation of alerts or notifications about their health metrics might inadvertently induce stress, which could have negative implications for their overall well-being, both mentally and physically.
3. *Aging-related stereotypes*: Some respondents expressed concerns about potential age-related perceptions surrounding the use of smart toilet seats. They feared that adopting such technology might inadvertently signal to others that they are managing severe chronic health conditions such as chronic kidney disease, urinary tract infections, gastrointestinal bleeding, constipation, or prostate cancer. This association, whether real or perceived, could reinforce negative stereotypes about the inevitable decline of health with age. However, it is essential to note that many older adults may embrace this age tech not necessarily because they manage specific health conditions, but as a proactive measure to maintain overall wellness and preemptively address potential health concerns.
4. *Privacy and security issues*: Respondents expressed reservations about data privacy and security associated with using intelligent toilet seats. They voiced their apprehensions regarding the nature of the data gathered, its sharing mechanisms, and potential access by unauthorized individuals. The inherent risks of internet-connected devices, such as potential data breaches and cyberattacks, further heightened their concerns. Given the sensitive nature of the data collected, many respondents indicated a preference for using devices that strictly adhere to Health Insurance Portability and Accountability Act guidelines. They emphasized the importance of ensuring that data are anonymized, not linked with personal identifiers or comprehensive health records, and not shared with third parties for nonconsented uses, such as research, to safeguard their privacy.
5. *Data quality*: Respondents expressed concerns about the accuracy and reliability of data gathered by the intelligent toilet seats. Although the device uses high-resolution optical scanning through lasers to identify potential health markers, the respondents questioned the consistent accuracy of these measurements. They voiced skepticism regarding the system’s ability to always capture valid wellness indicators, especially when foreign objects might be in the toilet. The potential for technical glitches that could lead to inaccurate readings, either false negatives or false positives, has also emerged as a concern. Furthermore, the inherent limitations and potential biases of AI and machine learning algorithms raised ethical apprehensions. Respondents emphasized that inaccuracies or biases in data might influence clinical decisions or treatments, underscoring the criticality of data precision in their willingness to adopt such technology.
6. *Internet outage risks*: Respondents highlighted concerns about potential internet disruptions and their impact on the functionality of intelligent toilet seats. Given that these devices rely on IoT technology for real-time data transmission, any lapse in internet connectivity could hinder the timely sharing of health data with medical professionals. They perceived that such interruptions could also impair communication and coordination between users and their health care teams, leading to the risk of conveying outdated or stale health parameters. As these sensing devices are dependent on an active internet connection to function effectively, any downtime could pause their key operations. Therefore, some respondents believed that the presence of a stable internet infrastructure, possibly supported by advancements such as 5G and robust Wi-Fi networks, would be crucial for the uninterrupted operation of such devices.

**Table 3:** List of constructs, definitions, anecdotal evidence, and count for perceived concerns and risks related to intelligent toilet seats.

Axial codes (constructs)	Definition	Sample quotes	Respondents, n
Embarrassment	The extent to which older people may feel uncomfortable and embarrassed by using intelligent toilet seats	“I will not feel OK when I am supposed to use it. It is embarrassing that a device is checking your private stuff to collect data.” “I get that it’s advanced technology, but there’s a certain level of privacy and dignity you want in the bathroom. I’d feel a bit exposed.”	83
Unnecessary concerns	The extent to which using intelligent toilet seats can raise excessive mental obsession	“I will feel obsessed all time since I will wait for a new alert saying that I am diagnosed with a serious infection that should be addressed ASAP.” “Every time it would analyze something, I’d be worried if I’m healthy or if there’s something wrong. Do I really need that daily stress?”	41
Aging-related stereotypes	The extent to which using intelligent toilet seats may create unfair and inaccurate attitudes that others hold about older people	“If I use it at home, my friends and guests will think I am using this because I am not feeling good, because I am old, and I cannot control myself.” “Just because we’re aging doesn’t mean we need a device for every little thing. I don’t like to become a symbol of dependency.”	38
Privacy and security risks	The extent to which using intelligent toilet seats can cause data security and privacy invasion concerns	“With the introduction of any new technology, there is always the risk of data breaches and malicious attacks that could invade data privacy.” “Based on all these hacking stories in the news, how can I be sure the information from the toilet seat remains confidential?”	124
Data quality risks	The extent to which using intelligent toilet seats can cause wrong, biased, and invalid clinical reports	“Human oversight and technical bias. AI systems can be subject to human bias and errors, which can lead to incorrect or unethical decisions.” “What if the toilet malfunctions and gives a false reading? A false alarm can be just as harmful.”	79
Internet outage risks	The extent to which the functionality of intelligent toilet seats can be disrupted due to network problems and internet outage	“This technology will not work if the internet connection is not stable. No internet means interruption, and the toilet seat system will be down.” “I’ve had my Wi-Fi go out plenty of times. If the toilet relies on that, it sounds like a struggle waiting to happen.”	45

Table 4 depicts the key themes signifying the main perceived concerns and risks of using intelligent toilet seats. The core themes were psychological concerns and technical performance risks. The psychological concern theme refers to uncomfortable feelings, unnecessary stress, and unfair and inaccurate attitudes of others toward aging owing to the use of smart toilet seats. Technical performance risks reflect possible technical malfunctions, inaccurate medical data, unsecured networks, and the potential threat of data breaches and unauthorized access.

**Table 4:** Selective codes representing perceived concerns and risks related to intelligent toilet seats.

Selective codes (themes)	Constructs involved	Definition
Psychological concerns	Embarrassment plus unnecessary concerns plus aging-related stereotypes	The extent to which older people may believe that using intelligent toilet seats may cause uncomfortable feelings, raise some unnecessary concerns, and develop stereotypes around them about aging
Technical performance risks	Privacy and security risks plus data quality concerns plus internet outage risks	The extent to which older people may believe that using intelligent toilet seats may cause possible technical risks, such as network vulnerability, unauthorized access, erroneous data, and malfunctions

## Discussion

### Theoretical Contributions

This qualitative study attempts to gain a deeper understanding of older adults’ adoption of a particular age tech. The research methodology adopted in this study focused on exploring older adults’ attitudes and perceptions regarding the use of intelligent toilet seats. Choosing a qualitative method to investigate older adults’ perceptions of intelligent toilet seats, despite their lack of direct experience, is crucial for capturing in-depth insights. Even if they have not used such technology, understanding older adults’ initial perceptions is invaluable; their viewpoints can highlight potential barriers to adoption, misconceptions, and design or educational improvements that vendors and developers might need to consider. Qualitative research delves deeper into perceptions, allowing for an open-ended exploration of initial reactions, apprehensions, and expectations. Given that this is potentially a first encounter with such technology for many older adults, this method captures the nuanced views shaped by cultural, generational, or personal beliefs. Although questionnaires provide quantifiable data, they might not grasp the breadth and depth of understanding qualitative approaches offer, especially when examining novel products or concepts.

Through survey interviews, the research aims to uncover the factors influencing older adults’ decision to adopt or reject this technology and the perceived benefits and challenges they might encounter with its use. This section explains the adoption facilitators and barriers that may shape opinions about this AI-powered technology and affect adoption decisions among older adults. Moreover, according to the results, we propose a guiding framework for future studies followed by practical implications and study limitations. It is worth noting that these theoretical implications and practical recommendations can be extended to a large extent to the broader realm of age tech, as smart toilet seats represent just one subset of this larger technological category.

### Adoption Facilitators

#### Health Care–Related Benefits

These were considered the most favorable advantages of using an intelligent toilet seat in this study. This result confirms that the key reason for using this intelligent system among older people is to improve health outcomes and enhance the quality of life. This is in line with previous studies on age tech adoption among the older population [68]. Building a smart environment through age tech and AI-powered devices can improve the quality of life, enhance safety, and provide timely health monitoring for older adults [69]. Similarly, AI-enabled screening tools can analyze toileting data to identify potential health issues, including conditions that may not be easily detectable through physical examinations or frequent visits. This technology can monitor excreta data, which holds valuable information about an individual’s health and alerts the individual and their health care provider to potential health concerns. Early detection of diseases leads to more prompt and effective treatment, improving health outcomes and quality of life for older adults [70]. It also increases the chances of survival and recovery for serious conditions such as cancer and allows more effective management of chronic conditions, such as diabetes.

Previous studies have indicated that older adults and their caregivers can efficiently obtain and integrate medical records and test results through seamless access to clinical reports [71]. Having complete and current clinical information enables health care providers to make informed decisions and provide better care to older adults [72]. Furthermore, seamless access to clinical reports facilitates improved communication and coordination among health care providers, ensuring that older adults receive consistent and effective treatment. With electronic access to real-time clinical reports, health care providers can save time and reduce the need for manual recordkeeping and duplicative tests, improving efficiency in managing chronic conditions in older adults.

Toilet-based health monitoring tools using smart toilets could offer preventive home-based continuous health monitoring for older adults experiencing chronic issues such as diabetes, liver disorders, and kidney diseases [44]. Regular monitoring of health parameters, such as frequency and volume of excreta, can provide older adults with the information needed to effectively manage chronic conditions, such as diabetes and kidney problems. Continuous monitoring allows health care providers to quickly detect changes in older peoples’ health, leading to improved outcomes [73]. This proactive approach to health can increase older adults’ engagement in managing their chronic conditions. In addition, the large amount of health data generated from continuous monitoring can be analyzed to provide valuable data-driven insights into health trends and patterns.

#### Technology-Related Advantages

One common concern related to age tech among older adults is the potential risk it may pose to users, possibly leading to injuries [74]. For instance, previous studies have highlighted that technology may endanger users’ health owing to malfunction and unsafe parts. Thus, safety is an important consideration when designing age tech for older adults, particularly for those with physical challenges [18]. The whole concept of age tech is to empower older adults to maintain their independence and remain actively engaged in their communities while ensuring their safety, health, and well-being through technology-driven solutions [75]. Safe technology aims to minimize harm to individuals and be reasonably free from causing damage. Intelligent toilet seats are designed in a way that does not cause harm to older adults or other users. Even if the AI-based algorithm does not work properly, the system will not physically injure older people because of improper use or dangerous functionality. As this age tech is not complicated to use and no specific training is required, the risk of unintended consequences and unforeseen circumstances that may threaten the health of older adults is lower.

Previous studies have indicated that aging management technology can help older adults monitor their physical activity levels and health markers using innovative features [76]. Advanced sensors and scanners enable intelligent toilet seats to accurately check bowel movements, track toileting data, and generate personalized and sophisticated analyses of an individual’s health risk factors. AI algorithms can predict future trends and patterns based on the toileting habits of older adults, allowing for proactive and preventive measures to be taken [77]. These patterns and relationships may not be easily noticeable by humans. Moreover, continuous data analysis provides real-time feedback. These unique features (ie, pattern recognition, predictive analysis, AI algorithm, machine learning, big data analytics, and real-time feedback) can offer new insights and opportunities to improve care for older adults [78].

#### Use-Related Benefits

As mentioned in previous studies, various types of age tech should be easy to use and require little to no training or instructions to operate [79]. The goal of ease of use is to minimize the frustration and confusion that older adults may experience when using age tech and to help them achieve their goals quickly and efficiently. The accessibility and usability of age tech can promote inclusivity and help to bridge the digital divide [12]. Smart toilets equipped with AI technology seemed to be free of effort and self-explanatory to the respondents of this study. This study also confirms the importance of the property of a technology or system that makes it simple and intuitive to operate and understand. The higher the user-friendliness level and accessibility of technology, the more likely it is that older adults would adopt it. Moreover, another aspect of usability is that different individuals or multiple people (of a family or in a nursing home) can share the same AI-based toilet seat. This technology is sharable but can be customized to meet the needs of different users. The data were collected from the toileting logs of each user and analyzed without interfering with the results of other users.

As highlighted in prior research, a common reason older adults may incorporate age tech in their daily activities is health care cost saving [75]. Costs because of medication mismanagement (especially for older adults with chronic health problems) are an integral part of their monthly expenditure [80]. Older people use age tech to reduce travel costs to and from physician’s appointments and costs related to receiving care planning and various treatment options. Using AI-based toilet seats can reduce the need for more expensive treatments, unnecessary medical tests, additional medications, and hospitalization through early detection and treatment of diseases that can prevent or delay the condition’s progression. Thus, older adults can save money on health care costs, and the health care system can also reduce the burden of more costly treatments and hospital stays.

### Adoption Barriers

#### Psychological Concerns

Emotional, ethical, and mental health issues arising from using technology have been highlighted in previous studies [81]. Age tech use can contribute to feelings of anxiety and stress, particularly when it involves detecting abnormalities in health parameters and the pressure to receive undesirable reports. The shadow of receiving alerts or alarms implying that older adults have been diagnosed with a difficult health issue can make them stressed, decrease their attention span, and generate unnecessary concerns [82]. Older adults may also feel embossed owing to the manner of data collection by AI-based seats as they monitor older people through excreta data. Consistent with previous studies, respondents repeatedly highlighted the concept of unobtrusively monitoring wellness parameters [76], as they believed that continuous monitoring of an older adult’s physical and emotional well-being should be conducted without causing discomfort or disruption. Thus, age tech collection practices, monitoring, and providing early warning signs of possible health issues should be in a manner that respects ethics, human rights, and the dignity of older adults.

Another source of stress and discomfort from using AI-based toilet seats is age-related stereotypes, which have been examined in previous studies [83]. Older adults may feel the stigma associated with using intelligent toilet seats as others (family or friends) would relate this need to aging and infirm health conditions. They may feel that using this technology is seen as a sign of weakness and dependence, or that it is only for older people with serious health problems. However, many older adults may use this technology only for prevention or early detection of diseases, not because they currently experience a serious infection. Previous studies indicate that perceived stigma can harm the dignity and self-esteem of older adults by making them feel ashamed of their use of technology [84]. It can also prevent older adults from embracing age tech and taking advantage of its benefits.

#### Technical Performance Risks

As highlighted in previous studies, concerns about potential technical issues are integral to using AI-based devices in health care [85]. Technical concerns can arise because AI algorithms embedded in age tech may produce inaccurate results or make incorrect decisions, primarily if they are based on faulty data or trained on limited, outdated, or biased data sets [86]. Older adults might be concerned about the quality and reliability of collected data, as sensors and AI algorithms mismeasure wellness parameters; all further analyses will also be flawed. As intelligent toilet seats send toileting logs over the internet to a central database, similar to other AI-based devices, they can be susceptible to hacking or other security threats, compromising sensitive information or disrupting their operation. Intelligent toilet seats function over the internet, and the probability of transmitting real-time data is low when the internet connection is poor. In this case, it is plausible that the system misses part of the data that could be very valuable for diagnosis and choosing treatment options. In addition, similar to other AI-powered tools, using AI algorithms in intelligent toilet seats can raise concerns about privacy and the protection of personal data [87], as AI systems may collect, store, and analyze vast amounts of information about older adults.

### A Proposed Guiding Framework

**Figure 1:**
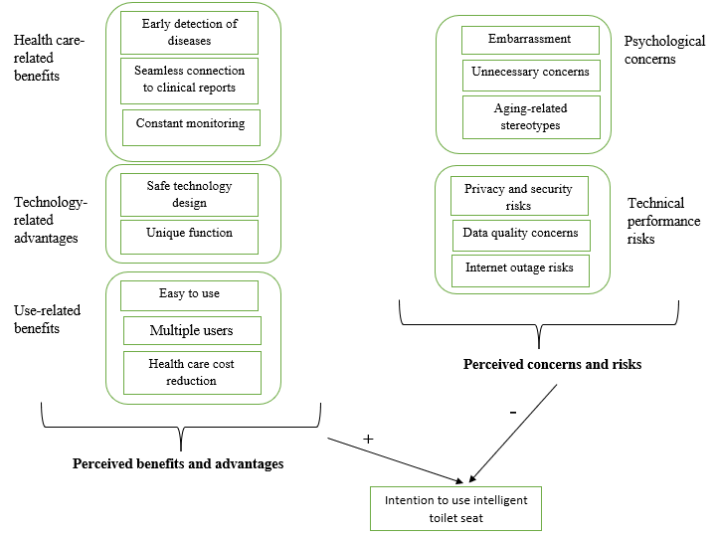
Guiding framework.

The results indicate that older adults expect several benefits from using intelligent toilet seats; however, concerns and risks are not negligible. The intention to adopt these AI-based toilet seats among older adults depends on comparing perceived positive and negative opinions associated with the system. Thus, if AI-powered toilet seats deliver less added value to older adults, they will negatively favor integrating the system into their daily lives. The guiding framework outlines the key variables, relationships, and mechanisms that are expected to exist in the research phenomenon being studied. This study contributes to the current discussion on using AI-powered systems in health care by providing a better picture of the potential benefits and possible concerns that may shape older adults’ perceptions about using intelligent toilet seats. Figure 1 presents a guiding framework illustrating the primary themes, categories, and concepts derived from the survey interviews.

### Practical Suggestions

On the basis of the current state of research in age tech and our findings of older adults’ perceptions and adoption of intelligent toilet seats, this study offers several practical recommendations and effective interventions that address both the facilitators and barriers to adoption, aiming to enhance the acceptance and use of AI-powered tools within the aging population.

1. *Increasing awareness and understanding*: Developers of AI-based toilet seats need to increase awareness and understanding of age technology through education and training programs for potential users focusing on its benefits and safe use. Educating older adults on the potential benefits of age tech can provide open and honest conversations about it on vendors’ websites, such as transparent, frequently asked questions, or web-based forums.
2. *Keeping technology accessible and easy to use*: Many older adults have physical conditions or health issues that make it difficult to use new technologies. Developers need to make intelligent toilet seats accessible and user-friendly, for example, by offering simple-to-use interfaces with little or no instructions, uncomplicated interactions with technology, and safe design. Moreover, integrating this age tech into health care services, such as telehealth and remote monitoring, can increase its accessibility and perceived value and make it more acceptable and accessible to older adults.
3. *Encouraging social support and reducing stigma*: To protect the dignity of older adults, it is essential to address the stigma surrounding technology use. One plausible way is to promote social support networks, such as family and friends, to encourage and assist older adults in adopting intelligent toilet seats. Educating older adults on the benefits of this technology is necessary via marketing efforts to alleviate the stigma associated with intelligent toilet seats. Encouraging them to try it and see how it can improve their daily life can also help to reduce stigma. It is also important to highlight that using this technology does not necessarily mean an older adult is dependent or weak. Instead, it can be seen as proactive and responsible, allowing individuals to care for their health and well-being.
4. *Offering tailored interventions and policies*: Developing tailored interventions specific to the needs and experiences of different older adult populations, such as those with chronic conditions or low income. Older adults may have different preferences and comfort levels with technology; therefore, assessing their needs and finding a solution that works best for them is crucial. For example, older adults with chronic issues may feel stressed, and receiving frequent warnings may exacerbate their uncomfortable experience with the system. Frequent alerts can be stressful and overwhelming for older adults with chronic illnesses. It is essential to find a balance between providing helpful reminders and notifications and avoiding adding to the stress and anxiety that older individuals may already be experiencing. One possible solution is to allow older adults to customize their notification settings and to choose the frequency and type of reminders they would like to receive. For example, some individuals may prefer to receive a daily summary of their health information. In contrast, others may choose to receive real-time notifications for critical events or changes in their condition. Moreover, using gentle and nonintrusive reminders could help AI-based toilet seat adoption be approached thoughtfully and compassionately. Another tailored example is the feasibility of using an automated bidet intervention to decrease physical assistance required from caregivers for toileting and toilet hygiene [88].
5. *Reassurance on system uptime*: Developers need to provide information about how intelligent toilet seats have been tested and validated and the measures in place to ensure their uptime. Some steps can be taken to mitigate the impact of internet outages. For instance, designing the seats with an offline mode so that they can continue to function even if the internet connection is lost. This could include storing data locally and syncing it with a remote server when the connection is restored. Moreover, seats can regularly back up the collected data to ensure that they are not lost during an internet outage. Thus, developing emergency protocols for internet outages should assure users that system downtime does not disrupt patient care.
6. *Monitoring and evaluating AI algorithms*: AI systems can sometimes make decisions that result in unintended consequences. Biases, discriminatory components, and errors may influence their mechanism in the data on which they are trained. The development of AI systems needs to prioritize safety, security, and ethical considerations. In addition, it is important to continuously monitor and evaluate their algorithms to identify and address potential risks. Thus, system designers must regularly check the effectiveness and safety of AI components embedded in intelligent toilet seats to promote positive health outcomes among older adults.
7. *Addressing privacy and security issues*: System designers need to be mindful of privacy and security concerns and the potential for misinterpretation of data. It is important to use AI algorithms that are based on reliable and unbiased data. In addition, developers must design AI systems that are secure, interoperable, and scalable to ensure that appropriate privacy and security measures are in place.
8. *Involving older adults*: Giving roles to older adults in selecting and setting up their age tech can help them embrace it faster. By providing older people with control over the system and helping them understand how intelligent toilet seats work to provide seamless connections to health care data, they are more likely to feel comfortable and confident using it. This policy can also help to build trust and increase technology adoption. Our results show that the best use case for implementing intelligent toilet seats seems to be in nursing homes. Nevertheless, it is worth noting that using this age tech is a personal choice and that older adults should not feel pressured to use it if uncomfortable. Everyone has the right to make decisions about their health and well-being, which should be respected and supported.

### Limitations and Directions for Future Studies

This qualitative research on older adults’ perceptions about toilet seats equipped with AI can provide valuable insights into their attitudes and perceptions, but it also has some limitations. First, the sample size of older adults in the study (n=174) may not be representative of the larger population of older adults in the United States, limiting the generalizability of the findings. It would be interesting for future studies to examine the perceptions of a larger sample of older adults. Second, older adults may have limited literacy on emerging technologies used in health care, which could impact their understanding of AI-powered technology and their perceptions of its use in health care. Future studies should focus on aging adults who are fairly familiar with age tech and AI-based systems in health care. Third, we used a web-based survey using MTurk as a recruitment method. This data collection method may introduce bias into the sample, as older adults who are more open to technology might be more likely to participate in the study. Thus, future research can extend this study by using other recruitment methods (such as in-person interviews) to examine a more representative sample. Fourth, although the insights from this study offer valuable perspectives on the acceptance and potential use of smart toilet seats by older adults, it is crucial to note that none of the respondents had first-hand experience with these devices before the study. As such, their feedback was based on perceptions rather than on actual use. For a more comprehensive understanding, future research should consider examining the experiences of individuals who have actively used smart toilet seats. This would directly assess the utility, challenges, and benefits associated with these innovative devices.

Fifth, the process of analyzing qualitative data can be challenging because it often involves coding and categorizing large amounts of unstructured data. In this study, 2 researchers familiar with age tech analyzed and coded the data. As qualitative studies rely heavily on the researcher’s interpretation of data, the researchers’ personal experiences with AI technology could influence their interpretation of the data and the findings. Future researchers can build upon this study by incorporating a wider range of perspectives and reducing researcher bias through collaboration with larger and more diverse research teams. This could result in identifying additional concepts, themes, or quotes that may have been overlooked in this study. Sixth, the respondents in this study have not raised the cost of using smart toilet seats. However, smart toilet seats with advanced biometric monitoring capabilities could cost approximately US $1000 plus installation fees, presenting a major financial barrier for most older adults. At this stage, there is little evidence that health insurance providers or medical practices are promoting smart toilets for in-home use. The high costs raise questions of how average consumers could realistically afford these devices. Further research is needed on potential subsidization or creative financing models to improve access to the potential benefits of smart toilets. Seventh, we did not focus on a particular smart toilet brand to gain a broader understanding of general user perceptions and attitudes toward the technology. Nonetheless, this could be an interesting area of research for future studies to delve deeper into brand-specific comparisons and their varied functionalities. Finally, this qualitative study identified key factors (perceived benefits and risks) influencing older adults’ intentions to use intelligent toilet seats. However, further quantitative research is required to fully understand the impact of these factors. This could involve examining the significance and importance of these constructs in implementing this technology in nursing homes. In addition, more studies are required to test and validate the relationships between the variables identified in the guiding model to determine the proposed model’s predictive power. However, despite these limitations, this qualitative research can provide important insights into the attitudes and adoption of intelligent toilet seats among the aging population and help inform the design and implementation of AI-based devices in health care.

### Conclusions

Previous studies indicate that age tech has the potential to greatly advance the lives of older adults by providing support, increasing independence, improving health care outcomes, and enhancing quality of life. For aging individuals and those with disabilities or chronic illnesses, smart toilets present an opportunity to live more independently while maintaining close monitoring of their health status. By sharing data and alerts with medical teams through telehealth platforms, smart toilets enable safer at-home living and can capture potential health issues early on. Their adoption stands to meaningfully improve the quality of life and health outcomes for susceptible populations. This study is an attempt to deeply explore, identify, and categorize aging adults’ perceptions of using a particular age tech, namely intelligent toilet seats. As the use of AI-based toilet seats for monitoring toileting logs is still a relatively new area of technology, more research is needed to determine the full range of benefits and potential privacy concerns. This qualitative study provides valuable insights into the opinions and attitudes of older adults toward the use of an intelligent toilet seat. The findings indicate a positive attitude toward using this age tech owing to health care benefits, technology-related advantages, and use benefits. However, the respondents also raised concerns about psychological distress and technological performance risks. It is crucial to identify ways to maximize the benefits of the technology while minimizing the risks it may pose to promote the widespread adoption of intelligent toilet seats among older adults. Addressing the barriers and concerns when developing technological solutions for older adults and designing technology based on their needs, expectations, and abilities can help them embrace toilet seats equipped with AI solutions. The results suggest a need for more education and awareness about the benefits (ie, early detection of diseases) and limitations of AI-based toilet seats, as well as increased efforts to address mental stress, concerns about the reliability of AI algorithms, and privacy and security concerns. Further research is needed to understand the broader impact of technology on older adults and to develop solutions that are both accessible and beneficial to this population. This research contributes to the field of age tech and assists in developing effective and user-friendly technological solutions for older adults.

## Appendix

Multimedia Appendix 1 Web-based survey.

## Abbreviations

Age tech: age technology
AI: artificial intelligence
ICC: intraclass correlation coefficient
IoT: Internet of Things
IRR: interrater reliability
MTurk: Mechanical Turk

## References

[1] Jakobsson E, Nygård L, Kottorp A, Malinowsky C (2019). Experiences from using eHealth in contact with health care among older adults with cognitive impairment. Scand J Caring Sci.

[2] Perissinotto C, Holt-Lunstad J, Periyakoil VS, Covinsky K (2019). A practical approach to assessing and mitigating loneliness and isolation in older adults. J Am Geriatr Soc.

[3] Etxeberria I, Etxebarria I, Urdaneta E (2018). Profiles in emotional aging: does age matter?. Aging Ment Health.

[4] Whelan S, Murphy K, Barrett E, Krusche C, Santorelli A, Casey D (2018). Factors affecting the acceptability of social robots by older adults including people with dementia or cognitive impairment: a literature review. Int J Soc Robot.

[5] Isabet B, Pino M, Lewis M, Benveniste S, Rigaud A-S (2021). Social telepresence robots: a narrative review of experiments involving older adults before and during the COVID-19 pandemic. Int J Environ Res Public Health.

[6] Sixsmith A (2020). COVID-19 and AgeTech. Qual Ageing Older Adults.

[7] Hakobyan L, Lumsden J, O'Sullivan D, Bartlett H (2013). Mobile assistive technologies for the visually impaired. Surv Ophthalmol.

[8] Dias D, Paulo Silva Cunha J (2018). Wearable health devices-vital sign monitoring, systems and technologies. Sensors (Basel).

[9] Yamagata C, Coppola JF, Kowtko M, Joyce S (2013). Mobile app development and usability research to help dementia and Alzheimer patients. Proceedings of the IEEE Long Island Systems, Applications and Technology Conference (LISAT).

[10] Chen Y-R, Schulz PJ (2016). The effect of information communication technology interventions on reducing social isolation in the elderly: a systematic review. J Med Internet Res.

[11] Malwade S, Abdul SS, Uddin M, Nursetyo AA, Fernandez-Luque L, Zhu XK, Cilliers L, Wong C-P, Bamidis P, Li Y-C (2018). Mobile and wearable technologies in healthcare for the ageing population. Comput Methods Programs Biomed.

[12] Shestakova NN, Djanelidze MG, Skvortsova MB (2022). AgeTech innovations as an instrument for older population inclusion. Proceedings of the 3rd International Scientific Conference on Sustainable Development (ESG 2022).

[13] Sixsmith A, Sixsmith J, Fang ML, Horst B (2020). AgeTech for cognitive health and dementia. AgeTech, Cognitive Health, and Dementia.

[14] Chu CH, Biss RK, Cooper L, Quan AM, Matulis H (2021). Exergaming platform for older adults residing in long-term care homes: user-centered design, development, and usability study. JMIR Serious Games.

[15] Oldenburg B, Taylor CB, O'Neil A, Cocker F, Cameron LD (2015). Using new technologies to improve the prevention and management of chronic conditions in populations. Annu Rev Public Health.

[16] Schicktanz S, Schweda M (2021). Aging 4.0? Rethinking the ethical framing of technology-assisted eldercare. Hist Philos Life Sci.

[17] Sharma M, Savage C, Nair M, Larsson I, Svedberg P, Nygren JM (2022). Artificial intelligence applications in health care practice: scoping review. J Med Internet Res.

[18] Sixsmith A, Rootman I, Edwards P, Levasseur M, Grunberg F (2021). AgeTech: technology-based solutions for aging societies. Promoting the Health of Older Adults: The Canadian Experience.

[19] Liu L, Daum C, Miguel Cruz A, Neubauer N, Perez H, Ríos Rincón A (2022). Ageing, technology, and health: advancing the concepts of autonomy and independence. Healthc Manage Forum.

[20] Yusif S, Soar J, Hafeez-Baig A (2016). Older people, assistive technologies, and the barriers to adoption: a systematic review. Int J Med Inform.

[21] Abadir PM, Chellappa R, Choudhry N, Demiris G, Ganesan D, Karlawish J, Li RM, Moore JH, Walston JD (2023). The promise of AI and technology to improve quality of life and care for older adults. Nat Aging.

[22] Anderson M, Perrin A (2017). Tech adoption climbs among older adults. Pew Research Center.

[23] Wang X, He Y, Zhang H (2023). How to influence behavioral intention toward age-friendly home modifications in urban older people aged 70. Gerontol Geriatr Med.

[24] Almathami HK, Win KT, Vlahu-Gjorgievska E (2020). Barriers and facilitators that influence telemedicine-based, real-time, online consultation at patients' homes: systematic literature review. J Med Internet Res.

[25] Molino M, Cortese CG, Ghislieri C (2021). Technology acceptance and leadership 4.0: a quali-quantitative study. Int J Environ Res Public Health.

[26] Winson A (2013). The Industrial Diet: The Degradation of Food and the Struggle for Healthy Eating.

[27] Kaushik K, Bhardwaj A, Dahiya S (2023). Smart home IoT forensics: current status, challenges, and future directions. Proceedings of the International Conference on Advancement in Computation & Computer Technologies (InCACCT).

[28] Global smart toilet market research report 2023. Valuates Reports.

[29] (2023). Smart toilet market size to reach USD 22.20 billion at a CAGR of 15.12% by 2030 - report by market research future (MRFR). Globe Newswire.

[30] (2022). Global smart toilet market size, share and industry trends analysis report by application (commercial and residential), by distribution channel (offline and online), by regional outlook and forecast, 2022 - 2028. Research and Markets.

[31] Armitage H (2020). ‘Smart toilet’ monitors for signs of disease. Stanford Medicine.

[32] Dorman A (2023). ‘Smart’ toilet seat tracks patients’ health, aids in diagnoses. McKnight's Senior Living.

[33] Ge TJ, Rahimzadeh VN, Mintz K, Park WG, Martinez-Martin N, Liao JC, Park S-M (2023). Passive monitoring by smart toilets for precision health. Sci Transl Med.

[34] Mace RA, Mattos MK, Vranceanu A-M (2022). Older adults can use technology: why healthcare professionals must overcome ageism in digital health. Transl Behav Med.

[35] Perdana A, Mokhtar IA (2022). Seniors' adoption of digital devices and virtual event platforms in Singapore during Covid-19. Technol Soc.

[36] Orlov L (2021). 10 barriers to boosting tech adoption by older adults in 2021. Aging and Health Technology Watch.

[37] (2022). Ageing and health. World Health Organization.

[38] Simpson RC (2005). Smart wheelchairs: a literature review. J Rehabil Res Dev.

[39] Pal D, Papasratorn B, Chutimaskul W, Funilkul S (2019). Embracing the smart-home revolution in Asia by the elderly: an end-user negative perception modeling. IEEE Access.

[40] Kurnianingsih K, Nugroho Le, Widyawan W, Lazuardi L, Prabuwono AS, Mantoro T (2018). Personalized adaptive system for elderly care in smart home using fuzzy inference system. Int J Pervasive Comput Commun.

[41] Borelli E, Paolini G, Antoniazzi F, Barbiroli M, Benassi F, Chesani F, Chiari L, Fantini M, Fuschini F, Galassi A, Giacobone GA, Imbesi S, Licciardello M, Loreti D, Marchi M, Masotti D, Mello P, Mellone S, Mincolelli G, Raffaelli C, Roffia L, Salmon Cinotti T, Tacconi C, Tamburini P, Zoli M, Costanzo A (2019). HABITAT: an IoT solution for independent elderly. Sensors (Basel).

[42] Marques B, McIntosh J, Valera A, Gaddam A (2020). Innovative and assistive eHealth technologies for smart therapeutic and rehabilitation outdoor spaces for the elderly demographic. Multimodal Technol Interact.

[43] Park S-M, Won DD, Lee BJ, Escobedo D, Esteva A, Aalipour A, Ge TJ, Kim JH, Suh S, Choi EH, Lozano AX, Yao C, Bodapati S, Achterberg FB, Kim J, Park H, Choi Y, Kim WJ, Yu JH, Bhatt AM, Lee JK, Spitler R, Wang SX, Gambhir SS (2020). A mountable toilet system for personalized health monitoring via the analysis of excreta. Nat Biomed Eng.

[44] Tasoglu S (2022). Toilet-based continuous health monitoring using urine. Nat Rev Urol.

[45] Smart toilet market size by application (residential and commercial), by distribution channel (offline and online), regions, global industry analysis, share, growth, trends, and forecast 2023 to 2032. The Brainy Insights.

[46] Huang JJ, Yu SI, Syu HY (2012). Development of the smart toilet equipment with measurements of physiological parameters. Proceedings of the 9th International Conference on Ubiquitous Intelligence and Computing and 9th International Conference on Autonomic and Trusted Computing.

[47] Hermsen S, Verbiest V, Buijs M, Wentink E (2023). Perceived use cases, barriers, and requirements for a smart health-tracking toilet seat: qualitative focus group study. JMIR Hum Factors.

[48] Balaceanu C, Marcu I, Suciu G, Dantas C, Mayer P (2019). Developing a smart toilet system for ageing people and persons with disabilities. Proceedings of the 6th Conference on the Engineering of Computer Based Systems.

[49] Lumetzberger J, Mayer P, Kampel M, Panek P (2021). Smart toilet seat configuration for more autonomy using an AI-based 3D depth sensor. Proceedings of the 14th PErvasive Technologies Related to Assistive Environments Conference.

[50] Bae JH, Lee HK (2018). User health information analysis with a urine and feces separable smart toilet system. IEEE Access.

[51] Caldeira C, Nurain N, Heintzman AA, Molchan H, Caine K, Demiris G, Siek KA, Reeder B, Connelly K (2023). How do I compare to the other people?": older adults' perspectives on personal smart home data for self-management". Proc ACM Hum Comput Interact.

[52] Berg BL, Lune H (2011). Qualitative Research Methods for the Social Sciences.

[53] DiStaso MW, Bortree DS (2012). Multi-method analysis of transparency in social media practices: survey, interviews and content analysis. Public Relat Rev.

[54] Al-Salom P, Miller CJ (2017). The problem with online data collection: predicting invalid responding in undergraduate samples. Curr Psychol.

[55] Marge M, Banerjee S, Rudnicky AI (2010). Using the Amazon Mechanical Turk for transcription of spoken language. Proceedings of the IEEE International Conference on Acoustics, Speech and Signal Processing.

[56] Mellis AM, Bickel WK (2020). Mechanical Turk data collection in addiction research: utility, concerns and best practices. Addiction.

[57] Mortensen K, Hughes TL (2018). Comparing Amazon's Mechanical Turk platform to conventional data collection methods in the health and medical research literature. J Gen Intern Med.

[58] Zhang B, Gearhart S (2020). Collecting online survey data: a comparison of data quality among a commercial panel and MTurk. Surv Pract.

[59] Turner AM, Engelsma T, Taylor JO, Sharma RK, Demiris G (2020). Recruiting older adult participants through crowdsourcing platforms: Mechanical Turk versus prolific academic. AMIA Annu Symp Proc.

[60] Ogletree AM, Katz B (2021). How do older adults recruited using MTurk differ from those in a national probability sample?. Int J Aging Hum Dev.

[61] Shaw A, Hargittai E (2021). Do the online activities of Amazon Mechanical Turk workers mirror those of the general population? A comparison of two survey samples. Int J Commun.

[62] Krippendorff K (2019). Content Analysis: An Introduction to Its Methodology.

[63] Blair E (2015). A reflexive exploration of two qualitative data coding techniques. J Methods Measure Soc Sci.

[64] McDonald N, Schoenebeck S, Forte A (2019). Reliability and inter-rater reliability in qualitative research: norms and guidelines for CSCW and HCI practice. Proc ACM Hum Comput Interact.

[65] Bornmann L, Mutz R, Daniel H-D (2010). A reliability-generalization study of journal peer reviews: a multilevel meta-analysis of inter-rater reliability and its determinants. PLoS One.

[66] Kwan G, Shaw JA, Murnane L (2019). Internet usage within healthcare: how college students use the internet to obtain health information. J Consum Health Internet.

[67] Finney Rutten LJ, Blake KD, Greenberg-Worisek AJ, Allen SV, Moser RP, Hesse BW (2019). Online health information seeking among US adults: measuring progress toward a healthy people 2020 objective. Public Health Rep.

[68] Demiris G, Hensel BK (2008). Technologies for an aging society: a systematic review of "smart home" applications. Yearb Med Inform.

[69] Bail K, Gibson D, Acharya P, Blackburn J, Kaak V, Kozlovskaia M, Turner M, Redley B (2022). Using health information technology in residential aged care homes: an integrative review to identify service and quality outcomes. Int J Med Inform.

[70] Ijaz N, Buta B, Xue Q-L, Mohess DT, Bushan A, Tran H, Batchelor W, deFilippi CR, Walston JD, Bandeen-Roche K, Forman DE, Resar JR, O'Connor CM, Gerstenblith G, Damluji AA (2022). Interventions for frailty among older adults with cardiovascular disease: JACC state-of-the-art review. J Am Coll Cardiol.

[71] Lawless MT, Marshall A, Mittinty MM, Harvey G (2020). What does integrated care mean from an older person's perspective? A scoping review. BMJ Open.

[72] Chen M, Decary M (2020). Artificial intelligence in healthcare: an essential guide for health leaders. Healthc Manage Forum.

[73] Lu L, Zhang J, Xie Y, Gao F, Xu S, Wu X, Ye Z (2020). Wearable health devices in health care: narrative systematic review. JMIR Mhealth Uhealth.

[74] Eby DW, Molnar LJ, Zhang L, St Louis RM, Zanier N, Kostyniuk LP, Stanciu S (2016). Use, perceptions, and benefits of automotive technologies among aging drivers. Inj Epidemiol.

[75] Rubeis G, Fang ML, Sixsmith A (2022). Equity in AgeTech for ageing well in technology-driven places: the role of social determinants in designing AI-based assistive technologies. Sci Eng Ethics.

[76] Czaja SJ, Boot WR, Charness N, Rogers WA (2019). Designing for Older Adults: Principles and Creative Human Factors Approaches, Third Edition.

[77] Fritz RL, Dermody G (2019). A nurse-driven method for developing artificial intelligence in "smart" homes for aging-in-place. Nurs Outlook.

[78] Li W, Chai Y, Khan F, Jan SR, Verma S, Menon VG, Li X, Kavita (2021). A comprehensive survey on machine learning-based big data analytics for IoT-enabled smart healthcare system. Mobile Netw Appl.

[79] Wang S, Bolling K, Mao W, Reichstadt J, Jeste D, Kim HC, Nebeker C (2019). Technology to support aging in place: older adults' perspectives. Healthcare (Basel).

[80] Mitzner TL, Boron JB, Fausset CB, Adams AE, Charness N, Czaja SJ, Dijkstra K, Fisk AD, Rogers WA, Sharit J (2010). Older adults talk technology: technology usage and attitudes. Comput Human Behav.

[81] Panico F, Cordasco G, Vogel C, Trojano L, Esposito A (2020). Ethical issues in assistive ambient living technologies for ageing well. Multimed Tools Appl.

[82] Abouzahra M, Ghasemaghaei M (2020). The antecedents and results of seniors’ use of activity tracking wearable devices. Health Policy Technol.

[83] Mariano J, Marques S, Ramos MR, Gerardo F, Cunha CL, Girenko A, Alexandersson J, Stree B, Lamanna M, Lorenzatto M, Mikkelsen LP, Bundgård-Jørgensen U, Rêgo S, de Vries H (2021). Too old for technology? Stereotype threat and technology use by older adults. Behav Inf Technol.

[84] Coghlan S, Waycott J, Lazar A, Neves BB (2021). Dignity, autonomy, and style of company: dimensions older adults consider for robot companions. Proc ACM Hum Comput Interact.

[85] Esmaeilzadeh P (2020). Use of AI-based tools for healthcare purposes: a survey study from consumers' perspectives. BMC Med Inform Decis Mak.

[86] Kelly CJ, Karthikesalingam A, Suleyman M, Corrado G, King D (2019). Key challenges for delivering clinical impact with artificial intelligence. BMC Med.

[87] Zhang Z, Shi Q, He T, Guo X, Dong B, Lee J, Lee C (2021). Artificial intelligence of toilet (AI-Toilet) for an integrated health monitoring system (IHMS) using smart triboelectric pressure sensors and image sensor. Nano Energy.

[88] Bollinger R, Somerville E, Keglovits M, Hu Y-L, Stark S (2021). Feasibility of an automated bidet intervention to decrease caregiver burden. Am J Occup Ther.

