# Supplementary material for: Older Adults’ Perceptions About Using Intelligent Toilet Seats Beyond Traditional Care: Web-Based Interview Survey
Source: JMIR Mhealth Uhealth. 2023 Dec 1;11:e46430. doi: 10.2196/46430 (PMC10724815; doi:10.2196/46430)
Supplement: Multimedia Appendix 1 [file mhealth_v11i1e46430_app1.docx]

**Appendix A- Online Survey:**

Dear participant, please read the following description.

Description of Smart Toilet Seats:

A smart toilet seat, often referred to as an "intelligent toilet seat," is an advanced bathroom fixture designed to enhance personal hygiene and monitor various health parameters through the analysis of waste matter.

Functionality:

Health Monitoring: The seat is equipped with sensors that can analyze excreta (both stools and urine) to detect potential health issues such as signs of dehydration, infections, or even chronic conditions like diabetes and monitor factors like dietary habits, hydration levels, heart rate, and warning signs for certain medical conditions. Many smart toilet seats come with features like bidet wash, warm air drying, and even self-cleaning capabilities.

Digital Integration: The seats connect to mobile apps via Bluetooth or WiFi to share data and insights. Data collected by the toilet seat can be sent to a paired smartphone app or a health dashboard, allowing users or healthcare professionals to monitor and evaluate health trends over time.

Multiple Users: multiple people can use the seat without interfering with each other's data. Also, if you have guests at home and want to disable data collection, you can simply push a button on the side of the smart seat to not collect their session information.

Appearance & Installation:

Look: These toilet seats look relatively similar to standard toilet seats but may have a slightly sleeker design containing internal sensors and additional side controls or a remote.

Installation: Installation is typically straightforward, similar to fitting a regular toilet seat. However, some models may require an electrical outlet for enhanced features.

Cost: The cost of a smart toilet seat can vary significantly based on its functionalities, brand, and region. On average, they can range from $250 to over $1000. Some advanced models with extensive health monitoring capabilities can be at the higher end of this range. Ongoing costs include replaceable parts and data plan fees for seats that transmit information to the cloud.

Before proceeding with the survey, please take a moment to familiarize yourself with the concept and functionality of smart toilet seats described above. Your understanding will be crucial in providing accurate and insightful responses.

**Questions**

**Demographics:**

- What is your age?

- What is your gender?

- What is your highest level of education?

- Do you have a chronic health condition? If yes, please name it.

- Do you live alone or with family/others?

- General Familiarity with AI and Awareness of Smart Devices:

1. Overall, how familiar are you with artificial intelligence (AI)- enabled devices?

2. Have you ever used a device that operates based on AI? (e.g., smart speakers, AI-based mobile apps, etc.)

3. If yes, please list the devices or tools you have used.

- General Familiarity with AI AI-powered devices and tools in healthcare

1. Overall , how familiar are you with AI-powered Tools in Healthcare?

2. Have you ever used AI-powered tools specifically for healthcare or health monitoring?

3. If yes, please specify which tools you have used and your experience with them.

- Familiarity with Smart Toilet Seats:

1. Have you heard of intelligent toilet seats before this survey?

2. If yes, where did you learn about them? (Options could include TV, Radio, Newspaper, Internet, Friends/Family, Doctor/Healthcare Professional)

3. Have you ever used a smart toilet seat?

**Perceived Benefits and Advantages:**

What benefits do you think an intelligent toilet seat can provide for older people like yourself?

How do you think such a device could improve your daily life or health monitoring?

**Perceived Concerns and Risks:**

Do you have any concerns or fears about using an intelligent toilet seat?

What factors would make you hesitant to use an intelligent toilet seat?

**Overall Opinions:**

What is your general opinion about integrating smart technology, like intelligent toilet seats, into the daily lives of older people?

Do you believe that intelligent toilet seats could be as trustworthy as traditional health monitoring methods?

How open would you be to having an intelligent toilet seat installed in your home bathroom?

**Willingness to Use:**

Would you be willing to use an intelligent toilet seat if one was made available to you? (Yes/No)

If no, what would be the main reason for your reluctance?

What would need to happen for you to feel comfortable adopting this type of technology?

**Barriers:**

What challenges do you anticipate in using an intelligent toilet seat?

What factors may prevent you from installing or using an intelligent toilet seat?

**Effectiveness of Interventions:**

If there were training sessions available on how to use intelligent toilet seats, would you attend? (Yes/No)

What kind of information would you need to feel encouraged about trying an intelligent toilet seat?

What would convince you that this technology is worthwhile and beneficial for you?
